# Supplementary figures and images for: Genetic variation for tolerance to pre-harvest sprouting in mungbean (Vigna radiata) genotypes
Source: PeerJ. 2024 Jul 23;12:e17609. doi: 10.7717/peerj.17609 (PMC11276771; doi:10.7717/peerj.17609)

**Supplementary Figure 1** Scree plot A) group I, B) group II, C) group III and D) overall
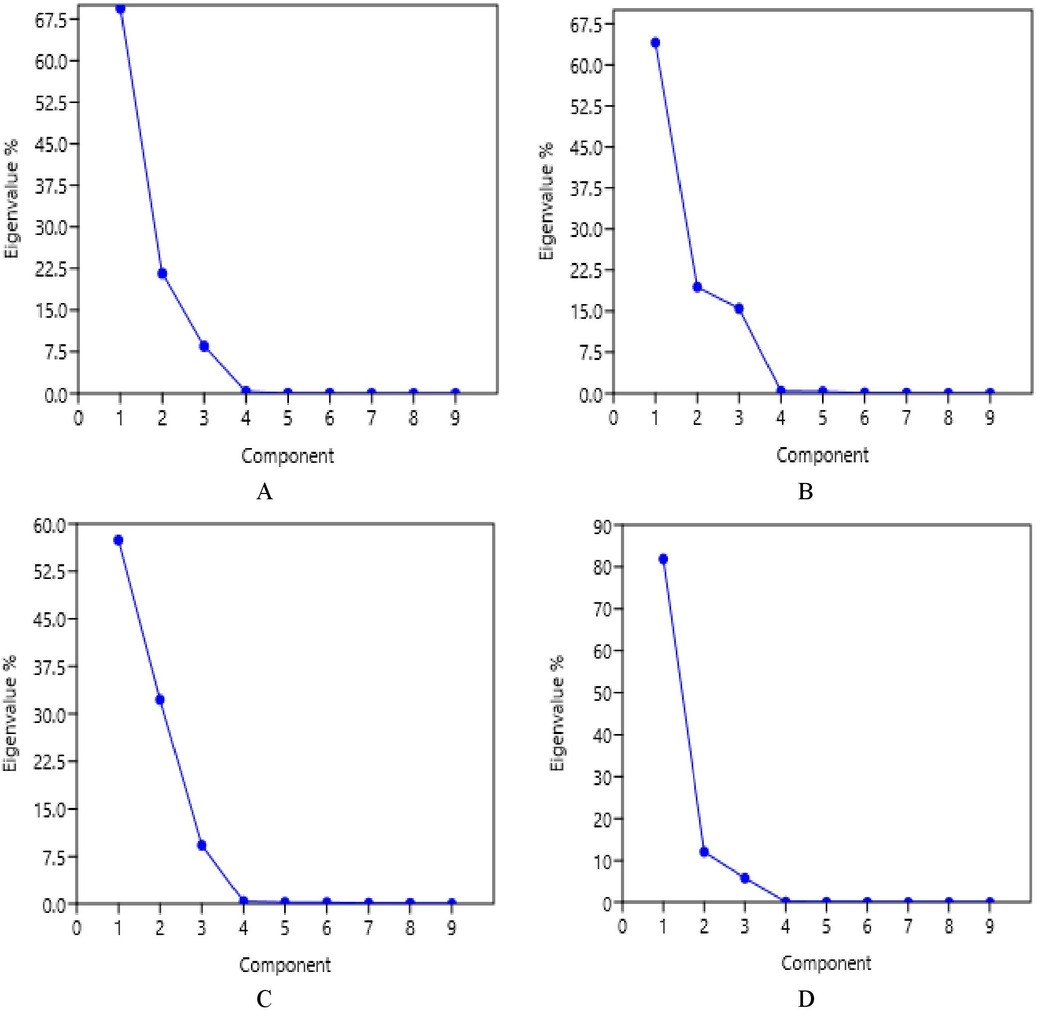

Supplement: Supplemental Information 2 [file peerj-12-17609-s002.docx]
