# Supplementary material for: Genetic variation for tolerance to pre-harvest sprouting in mungbean (Vigna radiata) genotypes
Source: PeerJ. 2024 Jul 23;12:e17609. doi: 10.7717/peerj.17609 (PMC11276771; doi:10.7717/peerj.17609)
Supplement: Supplemental Information 3 [file peerj-12-17609-s003.docx]

**Supplementary table 1** List of 83 mungbean genotypes along with their source.

| **Type** | **Genotypes** | **Source** |
| --- | --- | --- |
| **Released variety (23)** | BASANTI | Chaudhary Charan Singh Agricultural University (CCSAU), Hisar, India |
|  | MH 215 |  |
|  | MH 318 |  |
|  | MH 810 |  |
|  | MH 96-1 |  |
|  | Muskan |  |
|  | Satya |  |
|  | China Mung | Introduction from China |
|  | COGG 912 | Tamil Nadu Agricultural University (TNAU), Coimbatore, India |
|  | GANGA 1 | Agricultural Research Station, Sri Ganga Nagar, Rajasthan, India |
|  | HUM 2 | Banaras Hindu University (BHU), Varanasi, India |
|  | IPM 02-14 | Indian Institute of Pulses Research (IIPR), Kanpur, India |
|  | IPM 410-3 | Indian Institute of Pulses Research (IIPR), Kanpur, India |
|  | PDM 139 |  |
|  | LGG 460 | Andhra Pradesh Agricultural University (APAU), Lam, India |
|  | ML 818 | Punjab Agricultural University, Ludhiana, India |
|  | PS 16 | Indian Agricultural Research Institute (IARI), New Delhi, India |
|  | PUSA 0971 |  |
|  | Pusa Baisakhi |  |
|  | RMG 1028 | Rajasthan Agricultural Research Institute (RARI), Durgapura, India |
|  | RMG 991 |  |
|  | RMGP 1 |  |
|  | SML 668 | CSK Himachal Pradesh KrishiVishvavidyalaya, Palampur, India |
| **Advanced breeding lines (23)** | DMS 8 | Tirhut college of Agriculture, Dholi, Bihar, India |
|  | IPM 02-15 | Indian Institute of Pulses Research (IIPR), Kanpur, India |
|  | IPM 02-30 |  |
|  | IPM 409-4 |  |
|  | KM 16-58 | All India Coordinated Research Project (AICRP), MULLaRP, Kanpur, India |
|  | KM 16-69 |  |
|  | KM 16-76 |  |
|  | KM 16-81 |  |
|  | KM 16-82 |  |
|  | KM 2241 |  |
|  | KM 7-134 |  |
|  | MH 1442 | Chaudhary Charan Singh Agricultural University (CCSAU), Hisar, India |
|  | MH 565 |  |
|  | MH 934 |  |
|  | ML 1628 | Punjab Agricultural University, Ludhiana, India |
|  | PUSA 1131 | Indian Agricultural Research Institute (IARI), New Delhi, India |
|  | PUSA 1132 |  |
|  | PUSA 1332 |  |
|  | PUSA 1333 |  |
|  | PUSA 1341 |  |
|  | PUSA 1342 |  |
|  | PUSA 1441 |  |
|  | TM 9725 | Bhabha Atomic Research Centre (BARC), Mumbai, India |
| **Germplasm lines (37)** | IC 28083 | National Bureau of Plant Genetic Resources, New Delhi, India |
|  | IC 436637 |  |
|  | IC 436763 |  |
|  | IC 546476 |  |
|  | IC 546488 |  |
|  | M 1032 | Asian Vegetable Research and Development Centre (AVRDC), Taiwan |
|  | M 1053 |  |
|  | M 1131 |  |
|  | M 1156 |  |
|  | M 1168 |  |
|  | M 1255 |  |
|  | M 1358 |  |
|  | M 1370 |  |
|  | M 1372 |  |
|  | M 1378 |  |
|  | M 1400 |  |
|  | M 1421 |  |
|  | M 1447 | Indian Institute of Pulses Research (IIPR), Kanpur, India |
|  | M 1477 | Selection from Uttar Pradesh, India |
|  | M 1485 | Selection from Maharastra, India |
|  | M 204 | Selection from Bihar, India |
|  | M 145 | Indian Agricultural Research Institute (IARI), New Delhi, India |
|  | M 313 |  |
|  | M 422 |  |
|  | M 700 |  |
|  | M 684 | Selection from Hissar, India |
|  | M 703 | Asian Vegetable Research and Development Centre (AVRDC), Taiwan |
|  | M 837 |  |
|  | M 958 |  |
|  | M 981 |  |
|  | PLM 167 |  |
|  | PLM 271 |  |
|  | ML 1299 | Punjab Agricultural University, Ludhiana, India |
|  | ML 1451 |  |
|  | ML 1464 |  |
|  | ML 2037 |  |
|  | OLRM 4 | Orissa land race mungbean, Orissa, India |
